# Meeting of State Boards of Dental Examiners

**Published:** 1883-05

**Authors:** Geo. Cushing


ARTICLE III.

### MEETING OF STATE BOARDS OF DENTAL EXAMINERS.

LEXINGTON, Ky., Feb. 20, 1883.

In answer to a call issued by Dr. A. O. Rawls, F. H. Rehwinkel and A. W. Harlan, a committee appointed at an informal conference of the members of the various State Boards of Dental Examiners, held at Cincinnati at the time of the meeting of the American Dental Association, in August, 1882, the following gentlemen met at the office of Dr. A. O. Rawls, at this place, at 2:30 P. M. of this day: Drs. J. Taft, F. H. Rehwinkel and H. A. Smith, of the Ohio State Board of Examiners; A. W. Harlan and Geo. H. Cushing, of the Illinois State Board of Examiners; P. G. C. Hunt, of the Indiana State Board: Jos. Lewis, of the Vermont State Board; A. O. Rawls, of the Kentucky State Board; and Norman W. Kingsley, of the New York State Board.

Dr. Taft was chosen chairman and Geo. H. Cushing, secretary. Letters were read from Dr. Allen, Secretary of the Alabama State Board, and J. Hardman, President of the Iowa State Baard.

Drs. Rehwinkel, Rawls and Harlan were appointed a committee to arrange a programme, who reported the following:

"Your committee would make the following recommendations: 1st. That a permanent organization of the various State Boards be effected, to consist either of all the members of the Boards, or of delegates from the same, as the Association may determine. 2d. That a comparison of the laws of the different States be instituted, so that their differences and possible antagonisms may be reconciled in the draft of a law which shall embody the main features that experience has demonstrated to be essential, and that the draft so framed be recommended to those States desiring to secure such a law, for their adoption, or to those desiring to amend existing laws." This was accepted as a partial report.

After general dicussion, adjourned to meet at 7:30 this evening.

The conference met at 7:30 P. M., Dr. Taft in the chair. Dr. Kingsley read a letter from Dr. French, of the New York State Board.

The following committee was appointed to draft a law for consideration, which may be offered suggestively to those States desiring to secure a law, or to those who may desire to amend laws now in force—Drs. Cushing, Kingsley, and H. A. Smith.

The following were appointed a committee to draft a series of questions to be offered to the various State Boards as a basis for uniformity in the matter of examinations—Drs. Taft, Harlan and Rawls.

ADJOURNED TILL 10 A. M. WEDNESDAY.

Wednesday morning. Met at the office of Dr. Rawls at 10 o'clock, Dr. Taft in the chair. The committee appointed to draft a law made their report which was accepted, and upon motion was considered section by section, and finally adopted as a whole, as follows:

### AN ACT.

To insure the better education of practitioners of Dental Surgery, and to regulate the practice of dentistry in the State of ———

SECTION 1. Be it enacted by the people of the State of ———, represented in the General Assembly, that it shall be unlawful for any person who is not at the time of the passage of this Act engaged in the practice of dentistry in this State, to commence such practice unless he or she shall have obtained a license as hereinafter provided.

SEC. 2. A Board of Examiners to consist of five practicing dentists is hereby created, whose duty it shall be to carry out the purposes and enforce the provisions of this Act.

The members of said Board shall be appointed by the Governor, who shall select them from ten candidates whose names shall be furnished him by the ——— State Dental Society.

Three members, at least, of this Board shall be members of the ——— State Dental Society.

SEC. 3. Said Board shall choose one of its members President, and one the Secretary thereof, and it shall meet at least once in each year, and as much oftener and at such times and places as it may deem necessary. A majority of said Board shall at all times constitute a quorum, and the proceedings thereof shall at all reasonable times be open to public inspection.

SEC. 4. Within six months of the date of the passage of this Act, it shall be the duty of every person who is now engaged in the practice of dentistry in this State, to cause his or her name and residence, or place of buisness, to be registered with said Board of Examiners, who shall keep a book for that purpose. The statement of every such person shall be verified under oath before a Notary Public or Justice of the Peace in such manner as may be prescribed by the Board of Examiners.

Every person who shall so register with said Board as a practitioner of dentistry may continue to practice the same as such, without incurring any of the liabilities or penalties provided in this Act, and shall pay to the Board of Examiners for such registration a fee of one dollar.

It shall be the duty of the Board of Examiners to forward to the County Clerk of each county in the State a certified list of the names of all persons residing in this county who have registered in accordance with the provisions of this Act, and it shall be the duty of all County Clerks to register such names in a book to be kept for that purpose.

SEC. 5. Any and all persons, who shall so desire, may appear before said Board at any of its regular meetings and be examined with reference to this knowledge and skill in dental surgery, and if the examination of any such person or persons shall prove satisfactorily to said Board, the Board of Examiners shall issue to such persons as they shall find from such examination to possess the requisite qualifications, a license to practice dentistry in accordance with the provisions of this act. But said Board shall at all times issue a license to any person legitimately holding a diploma from any reputable dental college, upon his or her furnishing evidence satisfactory to the Board, of his or her right to the same, and upon the payment by said holder of a diploma to the said Board, of a fee of five dollars. All licenses issued by said Board shall be signed by the members thereof, and be attested by its President and Secretary; and such license shall be "prima facie" evidence of the right of the holder to practice dentistry in the State of ———.

SEC. 6. Any person who shall violate any of the provisions of this Act shall be liable to prosecution before any court of competent jurisdiction, upon information or by indictment, and upon conviction may be fined not less than fifty dollars, nor more than two hundred dollars for each and every offense. All fines recovered under this Act shall be paid into the common school fund of the county in which such conviction takes place.

SEC. 7. In order to provide the means for carrying out and maintaining the provisions of this act, the said Board of Examiners may charge each person applying to or appearing before them for examination for license to practice dentistry, a fee of ten dollars, which fee shall in no case be returned, and out of the funds coming into the possession of the Board from the fees so charged, the members of said Board may receive as compensation the sum of five dollars for each day actually engaged in the duties of their office and all legitimate and necessary expenses incurred in attending the meetings of said Board. Said expenses shall be paid from the fees and penalties received by the Board under the provisions of this Act. And no part of the salary or other expenses of the Board shall ever be paid out of the State treasury. All moneys received in excess of said per diem allowance, and other expenses above provided for, shall be held by the secretary of said Board as a special fund for meeting the expenses of said Board, he giving such bond as the Board shall from time to time direct. And said Board shall make an annual report of its proceedings to the Governor, by the fifteenth of December of each year, together with an account of all moneys received and disbursed by them pursuant to this Act.

SEC. 8. Any person who shall be licensed by said Board to practice dentistry shall cause his or her license to be registered with the County Clerk of any county or counties in in which such person may desire to engage in the practice of dentistry, and the county clerks of the several counties in this State shall charge for resistering such license a fee of twenty-five cents for each registration.

Any failure, neglect or refusal on the part of any person holding such license to register the same with County Clerk as above directed, for a period of six months, shall work a forfeiture of the license, and no license, when once forfeited, shall be restored, except upon the payment to the said Board of Examiners of the sum of twenty-five dollars as a penalty for such neglect, failure or refusal.

SEC. 9. Any person who shall knowingly and falsely claim or pretend to have or hold a certificate of license, diploma or degree, granted by any society, organized under and pursuant to the provisions of this act, or who shall falsely and with intent to deceive the public, claim or pretend to be a graduate from any incorporated dental college, not being such graduate, shall be deemed guilty of a misdemeanor.

The following explanatory note was adopted :

In presenting this draft of a law the committee would say that it is the result of the conference of those having had more or less experience in the matter of procuring such legislation and of the working of such laws, and it embraces the principal features which experience has demonstrated to be essential. It is offered suggestively, and not with the expectation that its phraseology will necessarily be adopted, or will be considered the best or most desirable that could be used—and of course each State seeking to secure such a law would do so under the direction of its own legal adviser, but the committee believe the features of vital importance for such a bill are embodied in the draft herewith submitted.

The following report of the committee on permanent organization was adopted.

Your committee on permanent organization respectfully report that after due consideration they would recommend that a permanent organization be effected, and that the Secretary of this meeting be instructed to notify the different State Boards of our action, and to urge them to either meet in corpora, or to send delegates to a meeting for the purpose of perfecting a permanent organization to be held on Monday, August 6, 1883, at 2 P. M. at the Cataract House, Niagara Falls.

It was voted that the proceedings of this meeting be furnished to the various dental journals for publication.

The committee appointed to draft questions for recommendation to the different Boards of Examiners, made a partial report which was adopted, and the committee was authorized to complete the series of questions and forward copies of the same to the different State Boards, with the recommendation, that they be used at the next examination, or that they be accepted as a minimum standard upon which to frame other questions.

On motion it was unanimously resolved, that this conference recommend to the various State boards that they adopt a rule to the effect that they will not accept the certificates of examination of other States, inasmuch as there are several States in which the law absolutely prohibits such recognition, which rule will tend to establish that uniformity of practice to desirable to be attained.

The proposition of Dr. Taft to do the necessary printing, leaving the matter of defraying the expenses to be settled at the next meeting, was gratefully accepted.

On motion of Dr. Smith it was unanimously voted that the thanks of this conference be extended to Dr. A. O. Rawls for his generous hospitality, which has made our visit one of great enjoyment.

Adopted by a rising vote.

On motion, adjourned to meet at the Cataract House, at Niagara Falls, on Monday, August 6, 1883, at 2 o'clock P. M.

GEO. CUSHING, Secretary.

